# Correction to “Gallium‐Doped MXene Nanozymes Protect Liver Through Multi‐Death Pathway Blockade and Hepatocyte Regeneration”

**DOI:** 10.1002/advs.75707

**Published:** 2026-05-15

Xiaopeng Cai, Jingwen Deng, Liqing Wang, Junjie Su, Dongyi Xian, Yilang Yan, Xinyu Yang, Chuanbin Wu, Tingting Peng, Yuan Ding, Guilan Quan*, Weilin Wang*, Min Zhou*, Chao Lu*, “Gallium‐Doped MXene Nanozymes Protect Liver Through Multi‐Death Pathway Blockade and Hepatocyte Regeneration,” *Advanced Science* 13, no. 14 (2026): e09079, https://doi.org/10.1002/advs.202509079.

In the originally published Figure 7C, the H&E‐stained image of the kidney after 8 h of V_2_C treatment was inadvertently duplicated and erroneously used to represent the 7‐day V_2_C treatment time point. The corrected image is presented below.


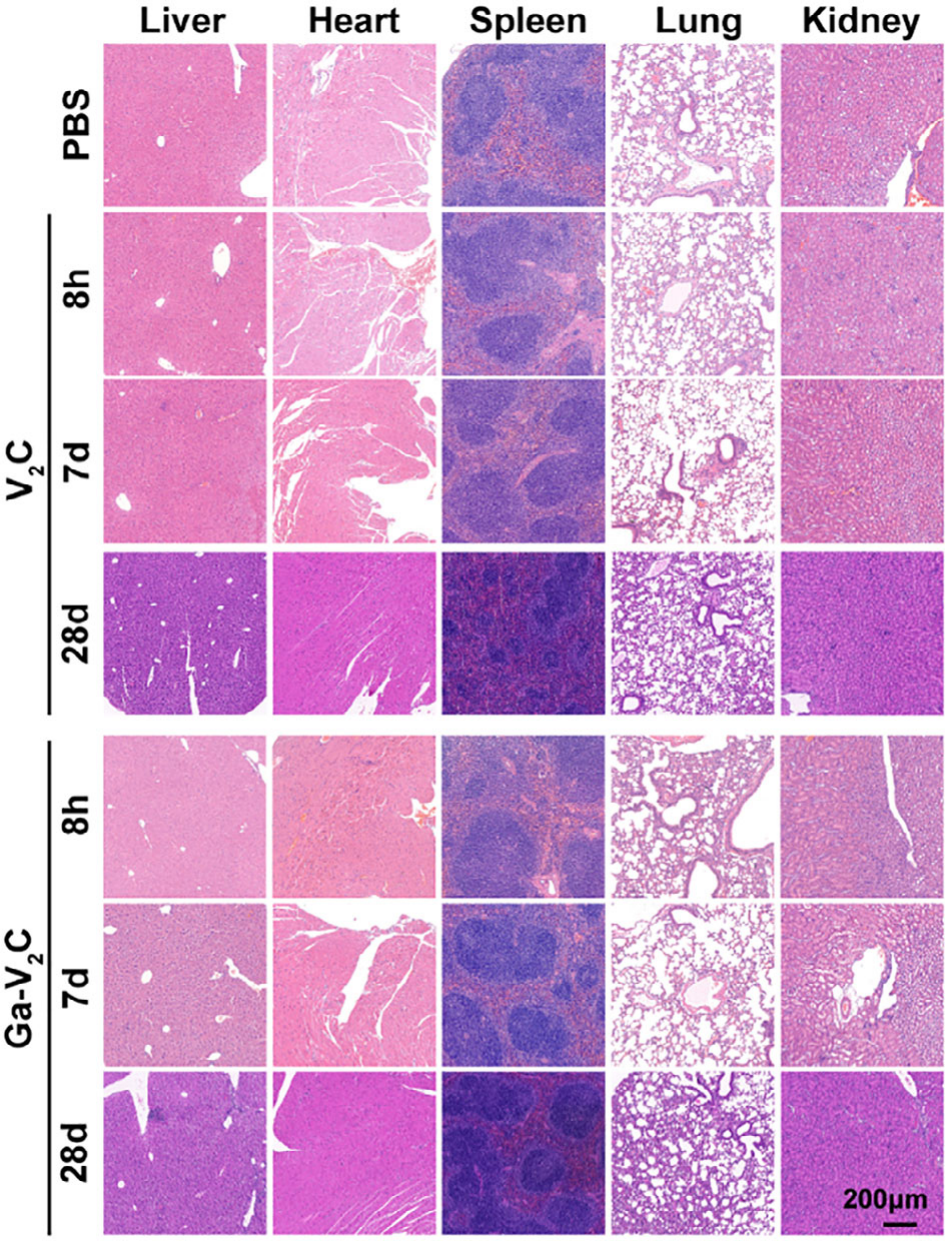
 **Corrected Figure 7C**. H&E staining of major organs was evaluated after 8 h, 7 days, and 28 days of indicated treatments.

The errors do not affect the original findings and conclusions. We apologize for this error.

